# A multivariate analysis of the impact of knowledge and relationships on perceptions about aging among generation Z—a starting point for public health strategies

**DOI:** 10.3389/fpubh.2025.1522078

**Published:** 2025-03-12

**Authors:** Silvia Puiu, Mihaela Tinca Udriștioiu, Mihaela Zăvăleanu

**Affiliations:** ^1^Department of Management, Marketing and Business Administration, Faculty of Economics and Business Administration, University of Craiova, Craiova, Romania; ^2^Department of Physics, Faculty of Sciences, University of Craiova, Craiova, Romania; ^3^Department of Kinetotherapy and Sport Medicine, Faculty of Physical Education and Sport, University of Craiova, Craiova, Romania

**Keywords:** perceptions, aging, generation Z, public health, thoughts, feelings, healthy habits, relationships

## Abstract

**Introduction:**

The main objective of the study is to better understand how knowledge about the natural process of aging and its implications, as well as the relationships with old people, shapes the perceptions of generation Z toward the meaning of old age.

**Methods:**

The research methodology we used is partial least squares structural equation modeling in which we developed a model with five variables: thoughts about aging, feelings about aging, healthy habits, knowledge about aging and relationship with old people.

**Results:**

The results revealed the direct and positive impact of both knowledge and relationships on the thoughts and beliefs of generation Z.

**Discussion:**

The findings are useful for managers in the public sector responsible for shaping more efficient health strategies meant to normalize aging and prepare young individuals for healthy aging.

## 1 Introduction

Aging is a natural process with many changes occurring in the body (1), but there are many factors that influence the way people perceive old age and old people. In this study, we focus on perceptions among generation Z because identifying earlier their cognitive patterns and beliefs can be useful in better understanding the way they live their lives and how they get old. Several studies show that with age, there is a decline in behavioral changes because of the routines people create in their lives, which makes them feel more comfortable (2, 3). This can be a starting point in shaping better health strategies addressed to the general population and meant to raise awareness on age-related challenges and increase the chances of having a population that maintains their health into old age.

Even if aging cannot be prevented, there are many things a person can incorporate into their lives in order to increase their health span (4). National Institute on Aging (4) emphasizes the need for research on aging because the process, even if it is natural, can be delayed, diseases can be prevented and we can age gracefully and in a healthy way. According to the World Health Organization (5), “healthy ageing is about creating the environments and opportunities that enable people to be and do what they value throughout their lives.” It is important to live many years, but it is more important to enjoy your life in good health during old age. In the end, what scares people is the fact that they will not be functional and they will get sick.

World Health Organization (6) highlights that behavioral changes are needed in order to be healthier, but these are not sufficient if they are not followed by a change in the way we think about aging and old people. National Institute on Aging (7) appreciates that physical, mental and cognitive health are equally important while also pointing to the misconceptions that people have regarding aging and the challenges they deal with (8). Healthy foods (9), exercise (10), sleep, socializing, learning new things, meditating to reduce stress, and having a hobby are things people can do in order to be healthier for a longer time.

McMaughan et al. (11) state that both “socio-economic status and access to healthcare” are factors that influence healthy aging. Buyl et al. (12) consider that “healthy aging is a contemporary challenge” and mention the fact that many studies found a good correlation between using digital services for health and other specific devices (e-health) and the users’ health. Considering the fact that Generation Z grew with technology, the chances of using such services and other technology for maintaining and improving health are higher. Generation Z includes young “people born between 1996 and 2010″ (13). Generation Z, who grew up with smartphones, gets bored if the information does not reach them by simply touching the smartphone or tablet screen with a finger or changing information quickly without reflecting too much on the content and retaining only the ideas that attract their attention (14). At the same time, generation Z represents a pivot population (102), being considered a “global consumer cohort and a driver of change for a sustainable future” (15). Generation Z has technology at “one’s fingertips” (16) and demonstrates similar consumer behaviors but with differences in perceptions (e.g., sustainable clothes, environment or business) compared to other generations (17). This generation prefers to use as a communication pattern the popularization or expression of ideas online (16, 18), which may lead to “digital disruption” (19) compared to previous generations.

Perceptions of aging among generation Z vary, but many youngsters believe in stereotypes when it comes to old people and what the aging process implies (20–22). Thus, a study on youngsters in Nigeria (21) (p. 439) revealed that they perceive elders as behaving “like children,” being “sickly, conservative, suspicious, and secretive.” Okoye and Obikeze (21) emphasize that this is due to the lack of education regarding aging. Omotosho and Tobiloba (22) interviewed youngsters about the way they see old people and found that many respondents (45%) perceive them as” difficult to please and too demanding,” “scary and old fashioned,” which made their interactions less frequent. The same negative attitude expressed as fear of aging is noted by Anderson and Gettings (20).

According to Chopik et al. (23), perceptions differ between age groups, with youngsters appreciating that old age starts after 50, while older people still consider themselves as young at this age. In their study, Bellingtier and Nguyen (24) found that adolescents registered a tendency toward perfectionism, which might explain their negative bias regarding aging. Also, the authors highlight that these beliefs and attitudes of younger generations can affect the relationships between generations, and if they do not change, they can affect these individuals as they themselves get old.

Not all studies reflect a bad view of the elders (25, 26), which might be explained by numerous factors such as the culture or the education received by youngsters in schools or their families. Gaspar de Almeida Santos and Nistor (25) interviewed Romanian young adults who expressed admiration for their grandparents and their role in their lives. Still, this positive attitude can be attributed to the fact that the interviews were conducted during the pandemic, and people were generally more willing to help elders in order to protect them from getting COVID-19. Perwaiz and Khan (26) emphasize the positive attitudes of young people in Pakistan toward old people who are appreciated for their wisdom. Lucăcel and Băban (27) show that the general negative attitude and perceptions of young adults toward seniors affect mutual relationships. The authors found that youngsters in their study perceive aging as “a bad thing that cannot be avoided” (27) (p. 151).

As Harmell et al. (28) mention, “aging is an enormous public health issue,” and they use the term “successful aging” or healthy aging. It is not sufficient to grow old, but to be healthy as long as possible. Thus, public health strategies are important in order to address these issues at a national level. Chodzko-Zajko and Schwingel (29) emphasize the need for “transnational strategies” to promote “active aging” so with a focus on sports activities. Another important aspect mentioned by the authors is that these strategies should be adjusted to each community’s socio-economic and cultural context because people are different and have different cognitive patterns, habits and perceptions of aging. Chen et al. (30) highlight the role of lifestyle in the health of the brain during old age, which shows the need for having better health strategies and “a vigorous public health effort to implement these strategies.” Naaldenberg et al. (31) conducted a study on the elders’ perceptions of what healthy aging means for them. This is a starting point for the local decision-makers when they create and implement strategies meant to support seniors. Marshall and Altpeter (32) appreciate the importance of the topic of aging for the leaders in the health sector who have both the power and the responsibility to implement strategies that help people live healthier lives.

It is vital for Romanian higher education institutions to actively engage with and understand generation Z’s particularities (33). From this perspective, academics should adapt their teaching methods and tools (including new technologies like artificial intelligence, virtual and augmented reality, customized software for different subjects and robots) to these particularities, making the content more attractive, accessible, interactive and engaging for students. In particular, understanding Romanian Generation Z’s ideas about aging and the older adult allows more targeted and specific interventions. In a formal environment, education should adapt itself to the particularities of generation Z. It should provide knowledge about the natural aging process with all its advantages and disadvantages based on curricular programs and offer innovative and technological support to generation Z. In an informal environment, education can cooperate with local communities and companies to offer knowledge on this topic through educational campaigns.

Mihai and Butiu (34) analyze Romanian family dynamics, where relationships with parents and relatives are preserved. Ion et al. (35) consider that this Eastern European culture is not well studied in terms of personality traits and that the multiple societal changes produced (bringing differences between “generations”) have exerted a minor influence on the distribution of personality traits in the general population. Vianello (36) analyzed the phenomenon of population migration from Romania to Italy and the care of the older adult left behind. He predicted that Romanian society would experience two new flows as migrants would either return to care for their parents or bring their parents to Italy to care for them.

Theoretical analysis by Leuțanu (37) reveals that the upbringing of children in a traditional and very conservative way in Romania is done in the family through the transgenerational transmission of values and moral-ethical aspects adapted to modern society’s values. The analysis of the grandparenting of Romanians is manifested in a variety of ways; according to Ducu (38), more data and analysis are needed to understand the dynamics of the process, especially at the transnational level. Many people chose to leave Romania, but the ties with the family were not lost. Ducu and Telegdi-Csetri (39) analyzed transnational family and grandchild-grandparent relationships. The communication gap between elders and youth in Romania is bridged because it “conveys a sense of Romanianness abroad and a cosmopolitan openness towards home” through various gifts (food or traditional products). This exchange, which works both ways regardless of the geographical location of the subjects, is somehow a symbolic way of nurturing the bond between generations, intergenerational care and feelings of love even if they are not close to each other. The authors also present another reality most visibly manifested between migrants and residents who manage to communicate virtually, bridging the distance and communication gap between generations and maintaining the flesh family identity at the transnational level.

## 2 Literature review

In our study, we focused on five variables related to aging and old people: thoughts, feelings, healthy habits, knowledge about aging and relationship with old people. Thoughts and feelings of Generation Z on aging influence many facets of their lives at present and in the future, as well as the way they interact with elders in their families and communities.

### 2.1 Knowledge about aging

Ginschel and Schlüter (40) focus on the role that schools and teachers need to play in order to change distorted beliefs and images of old age. Their study conducted on adolescents in Germany revealed that there is a deficit in the knowledge, especially regarding the biological processes happening in the body as we age and also on the diseases that can occur and the lifestyle changes that can be made to prevent or delay them. Allen Jr. (41) studied the impact of knowledge about aging among youngsters on their views of old people, and the results showed that they have a negatively biased image toward the elders. Langer (42) emphasize the role of aging education from an early age in order to help children have a more realistic and balanced view of aging and old people.

Generation Z more frequently accesses internet resources to learn health information than older people but demonstrates a lower ability and desire to be truly informed about health (43). Jia and Li (44) identified an inverse relationship between risk perception and health information avoidance behavior. Under social peer pressure and high expectations, young people are likely to avoid health risk information, the reason being stress avoidance. Cognitive and affective reasons underlying these behaviors are related to risk perception, conflicting values, information abundance, low confidence in information sources, anxiety, frustration driven by the outcomes found, and desire to remain positive. Ageism may also occur due to these behaviors characteristic of generation Z, as old age is stereotypically associated with illness, serious problems, suffering, increased needs and death.

O’Hanlon et al. (45) noticed that older students registered higher levels of both knowledge on aging and positive attitudes toward elders than younger students. Cooney et al. (46) identified that factors such as limited knowledge, lack of contact with old people and feelings such as “anxiety about aging” (p. 28) led to ageism and discrimination based on someone’s age. Yu and Chen (47) emphasize the need for specific programs meant to raise the level of knowledge about aging and the needs and challenges of old people in order to change biased attitudes and beliefs toward them.

Thus, the following hypotheses were developed:

*Hypothesis* 1 (H1): Knowledge about aging has a direct and positive impact on the healthy habits of generation Z.

*Hypothesis* 2 (H2): Knowledge about aging has a direct and positive impact on the relationships with old people in which generation Z is engaged.

### 2.2 Thoughts about aging

Thoughts about aging are beliefs, opinions, and perceptions formed in someone’s mind. They can be influenced by many aspects of the environment while also influencing the feelings, behavior and attitudes of that person (this dynamic is the foundation of the rational emotive behavior therapy initiated by Albert Ellis). Ageism is not only external, from youngsters toward elders, but also internal when old people themselves do not trust their capacity to do or try something new, generating defeatist and dysfunctional thoughts (48).

Keeling (49) studied rural communities in New Zealand and found close connections between children and their grandparents, which influenced their perceptions of aging. Another study on Generation Z grandchildren was conducted by Fruhauf et al. (50), who found that those caring for their grandparents developed a meaning for their behavior, mostly related to the duty they felt they had toward the elders and also in order to prepare “for the future” (p. 887).

The perceptions of young generations toward old people can be reflected in the way elders say (51) that they are treated by younger generations in their families when they do not understand technology and need help in this regard. The respondents in the study mentioned the frustration and lack of patience exhibited toward them when they needed more time to learn something new. Hegedüs et al. (52) emphasize that the negative thoughts about aging among young Hungarians might affect not only their relationship with old people but also their relationship with their future aging selves, which poses important risks to their mental health. The cognitive part of ageism translated into negative beliefs and perceptions can be changed by educating people on these issues and also increasing the high-quality contact between youngsters and elders (53), which changes both their feelings and behaviors. Generation Z accepts volunteering as a way to learn something new, improve communication, organize events, and do something useful for their community (54, 55). Regarding generation Z’s relationships, thoughts, and beliefs, some solutions exist at the family, education and decision-maker levels. Each family should be able to provide an optimal environment for interaction between children, parents and grandparents. Intergenerational relations can also be enhanced through more social entrepreneurial services.

The following hypothesis was developed: Hypothesis 3 (H3): Knowledge about aging has a direct and positive impact on the thoughts and beliefs of generation Z regarding aging as a process and old people.

### 2.3 Relationships with old people

The literature on the importance of relationships with old people is vast, many studies (25, 56–58) emphasizing the role intergenerational contact plays in the way we perceive the aging process. Gaspar de Almeida Santos and Nistor (25) point to the reciprocal benefits of contact between young and old generations because the former learn about aging in a more realistic way while the latter is engaged socially, which brings positive outcomes for their health. The authors also notice that “the link between these two age groups seems to be quite fragile” (25) (p. 24).

Verhage et al. (58) emphasize that the prior experience of youngsters with their grandparents influences their perceptions and image of aging when they interact with other elders. Park et al. (57) interviewed young people who spent at least a year with a grandparent and found that they better understood that aging comes not only with difficulties but also with gains. The respondents also reflected more on their future life as old people, feeling like they can follow the example set by a grandparent in terms of healthy aging or, on the contrary, that they are more prepared to prevent ailments that a grandparent struggled with. The authors note the need to offer young adults the possibility to interact with elders if they do not have grandparents close to them because relationships with old people help them navigate life in a more resilient way. Intergenerational contact is beneficial for younger and older adults (103). Another aspect emphasized by them is the role of education in aging for developing healthy perceptions (57).

Weiss andZhang (59) compared the views and stereotypes of six generational groups in the United States, China, and Germany, developed countries on three continents with different historical backgrounds and cultures. The results showed that older generations are a source of respect, and young people perceive them positively. However, the advanced older adult are perceived in a less favorable light by the younger generations, probably through the prism of illness and impending death.

According to Bontekoning (104), Generation Z in the Netherlands does not keep in touch with older people who are authoritarian, have rigorous habits, are overly conservative, lack flexibility, or do not give feedback on their actions. According to this author, for an older person to matter to a generation Z person, the latter would only have to accept the younger person’s comments, which is communication on the same social level, not based on age criteria.

Flamion et al. (56) show that discrimination and negative opinions regarding old people are a tendency that starts in childhood, especially in children who do not have close and positive contact with their grandparents. The authors found that boys had more negative perceptions toward elders than girls and that grandparents’ health influences the children’s perceptions.

The following hypothesis was developed: H4: Relationships with old people have a direct and positive impact on the thoughts and beliefs about aging held by generation Z.

### 2.4 Healthy habits

Healthy habits among younger generations are extensively studied, and they refer to eating, sports, sleep, and socialization. Kendig et al. (60) emphasize the need to have strategies meant to promote a healthy lifestyle because the results showed that this is a predictor for healthy aging. Fritsch et al. (61) note that “an active, engaged lifestyle… can have a positive impact on cognitive functioning in late life,” which is useful to understand in order to take measures earlier, not only by youngsters but also by those who develop public health strategies. Cheung and Dawkes (62) studied students’ healthy eating habits in a country known for the high life expectancy among both men and women (Hong Kong). Tasdemir-Ozdes et al. (63) researched both young and old people in the way they incorporated healthy habits into their lifestyles and found that this was mostly determined by the perspectives they had on their health in the future. As the authors say, “encouraging healthy choices” is the right decision for each individual (63) (p. 618).

“Cultural pressure and social identity” are perceived differently in the new digital society, penetrating young people’s lives through online platforms (18). Budi et al. (64) consider that online social media represents new tools for the young generation’s opinion-shaping and social mobilization. Budi et al. (64) consider that online social media represents new tools for the young generation’s opinion-shaping and social mobilization. There is a significant difference between health digitization and formal health education. Regarding health, Generation Z uses unconventional technology and internet-based information tools, such as social media platforms. Those working in the field of health education need to identify solutions that incorporate such tools and aid communication with this generation (43).

By directly observing the dynamic (self-reported) behaviors of health information used by Generation Z, the authors of a study conducted in Hungary analyzed health information-seeking behavior on the Internet. This study found no significant differences related to health information-seeking behavior between baby boomers and Generation Z, which provides a strong rationale for developing the health literacy skills of baby boomers through electronic means (65) and eventually, with the help of younger people around them. Another interesting point emphasized by this study is that those who use the Internet more often to search for health information rate their health status as lower. In their study, Dida et al. (66) noticed that adopting an attitude toward health information is influenced by the level of education and generation and less by the person’s gender.

### 2.5 Feelings about aging

Anderson and Gettings (20) show that youngsters in their study had “mixed feelings” toward aging, with more negative feelings (fear) expressed after the COVID-19 pandemic. The authors conclude that the way old people and aging are presented in media or the general communication in the community someone lives is a determining factor of how young people perceive the aging process and which also shapes their feelings. As other authors mentioned (52), these affect how they relate to elders and how they will perceive and feel toward their aging. Ayalon (67) studied the feelings of both young and old adults in European countries and found that “women and younger adults reported more favorable feelings toward younger relative to older adults” (p. 898), and the differences between countries were not significant, the authors pointing to the fact that European countries share more contextual similarities. Bousfield and Hutchison (68) studied the anxiety felt by students in contact with elders. They noticed that high anxiety was prevalent among those who had previous bad experiences or a lack of contact with old adults. Thus, their anxiety influenced the way they behaved in their interactions with elders. Aging-related anxiety is a universal fact; it manifests itself in acute spikes for each of us (69). The manifestations among generation Z are more visible, demonstrating a vulnerability. This topic, aging, seems to be the Achilles’ heel for young people. It seems to be a much too present concern in their mentality than for other generations. In 2024, there was a debate among generation Z exponents of digital media about how the passing of the years manifested among them. The overcritical phrase debated was that for generation Z, “ageing is like milk,” according to The New York Times (70). The idea is not new; the American actress Helen Hayes (who died at 93 in 1993) gave a well-known inspirational quote: “Age is not important unless you are a cheese.” She suggested that human beings are vulnerable in the face of time.

The following hypothesis was developed: H5: Thoughts about aging have a direct and positive impact on the feelings of generation Z about the aging process and old people.

## 3 Research methodology

**Figure 1:**
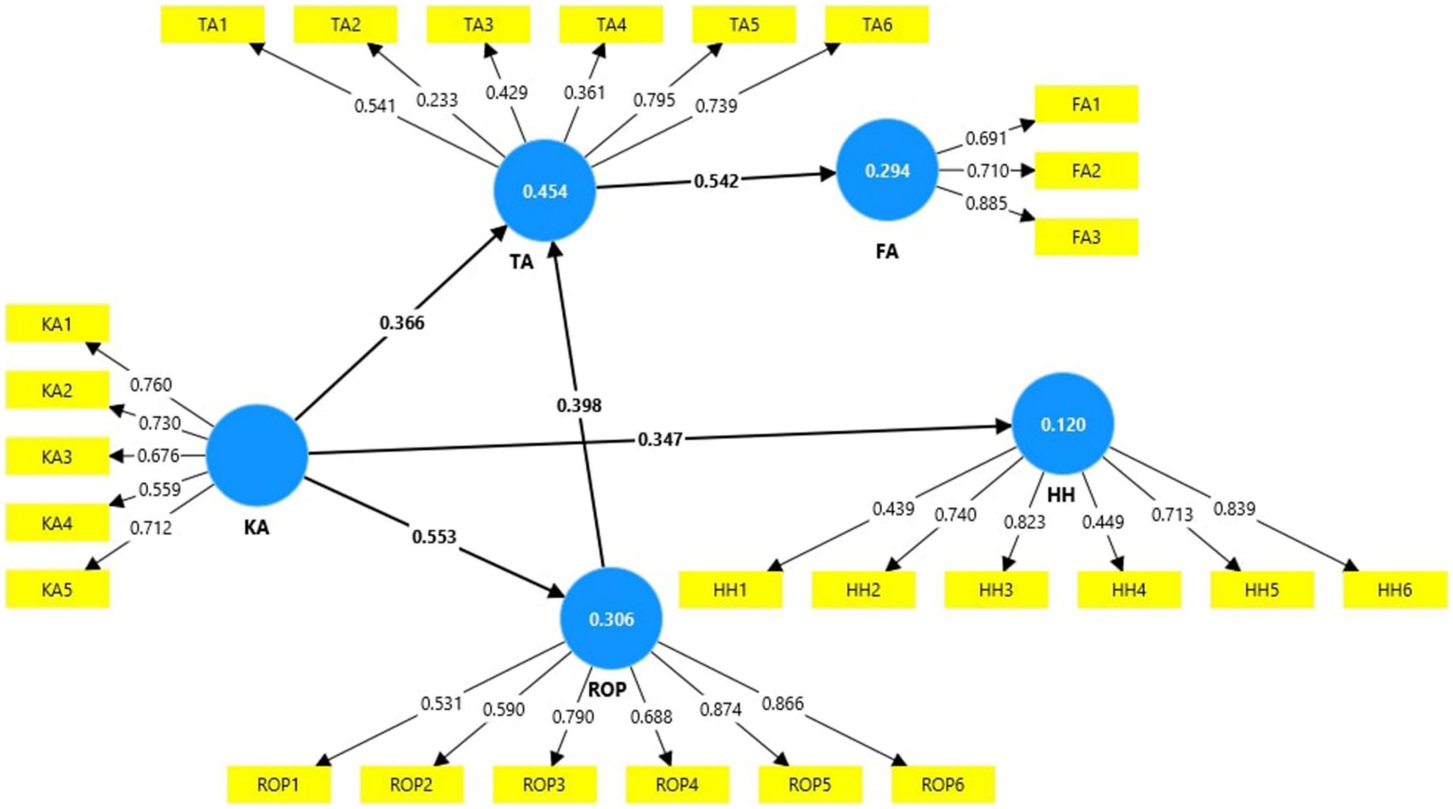
The research model.

Partial least squares structural equation modeling and SmartPLS version 4.1.0.0 (71) were used as a research methodology. The aim of this study was to better understand the impact of knowledge about aging and the relationships with old people on their thoughts regarding this natural process. We developed five hypotheses which link the variables in the research model. Figure 1 illustrates the model with the five variables, and Appendix A presents the model’s constructs and items. The use of PLS-SEM methodology and its robustness, applying the bootstrapping test and the method’s variance approach ensure the mitigation of outliers’ influence (72).

Further, we determined the outer loadings to ensure that we keep only the relevant items in the model (Table 1). Outer loadings higher than 0.7 show a high reliability of the items in the model (73–75), and those between 0.6 and 0.7 are considered to have an acceptable reliability (74, 76). The correlation matrix between the variables and the mean and standard deviation for each item kept in the model are presented in Table 2, the data being calculated in SmartPLS.

**Table 1:** Reliability and collinearity for the items in the model.

Items	Outer loadings	VIF
FA1	0.691	1.525
FA2	0.710	1.577
FA3	0.885	1.210
HH1	0.439	1.101
HH2	0.740	1.654
HH3	0.823	2.505
HH4	0.449	1.249
HH5	0.713	1.598
HH6	0.839	2.522
KA1	0.760	2.200
KA2	0.730	2.075
KA3	0.676	1.269
KA4	0.559	1.184
KA5	0.712	1.267
ROP1	0.531	1.198
ROP2	0.590	1.263
ROP3	0.790	2.093
ROP4	0.688	1.553
ROP5	0.874	3.250
ROP6	0.866	3.198
TA1	0.541	1.128
TA2	0.233	1.115
TA3	0.429	1.173
TA4	0.361	1.147
TA5	0.795	1.361
TA6	0.739	1.346

**Table 2:** Correlation matrix and descriptive statistics for items kept in the model.

Variables	Constructs	Mean	Standard Deviation	Skewness	Kurtosis	FA	HH	KA	ROP	TA
FA	FA1	3.19	0.729	−0.183	−1.020	1.000	0.153	0.504	0.594	0.550
	FA2	2.89	0.715	0.117	−1.105					
	FA3	3.76	0.452	−0.468	−0.669					
HH	HH2	2.78	0.659	0.316	−1.211	0.153	1.000	0.277	0.198	0.170
	HH3	3.13	0.485	−0.105	−0.642					
	HH5	3.67	0.713	−0.482	−0.682					
	HH6	3.24	0.505	−0.800	−0.007					
KA	KA1	3.94	0.669	−0.621	−0.392	0.504	0.277	1.000	0.507	0.512
	KA2	3.75	0.694	−0.175	−0.649					
	KA3	3.31	0.727	−0.013	−0.474					
	KA5	3.61	0.643	−0.393	−0.599					
ROP	ROP3	3.22	0.552	−0.057	−0.690	0.594	0.198	0.507	1.000	0.591
	ROP4	2.98	0.679	0.018	−0.905					
	ROP5	3.55	0.423	−0.126	−0.399					
	ROP6	3.54	0.432	−0.212	−0.317					
TA	TA5	3.82	0.457	1.501	1.692	0.550	0.170	0.512	0.591	1.000
	TA6	3.73	0.557	−0.414	−0.678					

Skewness and kurtosis in Table 2 are in normal range, between −2 and 2, showing a normal distribution of results. For outliers’ analysis, we used JASP software (101) and we applied Boxplots with Labels for outliers. There were no outliers for FA, HH, and KA. For ROP5 there are 7 outliers, for ROP6–9 outliers, and 6 outliers for TA5. The number of outliers is low and can be kept without affecting the model (77) which is also shown by the negative kurtosis in Table 2.

For conducting the single-source bias in SmartPLS (known in this software as common bias), we conducted the PLS-SEM algorithm to see the collinearity statists of the model, mainly the VIF values. As we can notice from Table 2, all of them are below 3.3, which is the value required for making sure that the research is not negatively affected by bias (78). Besides this, we ran a single factor test to check if the first factor creates common bias. Thus, we used JASP software to conduct exploratory factor analysis and we chose the single factor option, the results being presented in Table 3. The variance for the first factor is only 28.6% which shows that there are no common bias concerns involved.

**Table 3:** Exploratory factor analysis and single factor test for testing single-source bias.

		Unrotated solution		
	Eigenvalues	Sum Sq. loadings	Proportion var.	Cumulative
Factor 1	5.473	4.867	0.286	0.286

For non-response bias, we grouped respondents in early responders (those who answered in the first week) and late responders (those who answered after the first week). We sent our survey only once to potential respondents but we used timestamps to make the distinction between early responders (243 respondents) and late responders (19 respondents). We used JASP software to check for non-response bias. We used variables such as gender, environment, employment status to see if there are significant differences between the two groups. The results in Table 4 show that statistically there is no significant difference (p is above 0.05) between the early and late responders and thus, there is no bias revealed.

**Table 4:** Non-response test between early and late responders.

Independent samples T-test			
	*t*	df	*p*
Gender	1.582	260	0.115
Environment	0.316	260	0.752
Employment	−0.002	260	0.998

We used Google Forms to build the survey. The survey was structured in five parts, focusing on the hypothesis of this study and 26 statements. We sent the link to 600 individuals belonging to Generation Z via institutional email or Google Classroom. The survey contained an Informed consent. Participants were informed that any intervention would not follow the study and that they could not be identified directly or indirectly. Participation in this study was free; no incentives were offered. The data were collected between January and April 2024, and we received answers from 262 respondents. Besides the descriptive questions regarding the sample, we used the psychometric Likert scale (1-total disagreement to 5-total agreement) for the statements about aging included in the survey. The decision to use a questionnaire on the Likert scale was to nuance the respondents’ response options. The survey is a structured one (as shown in Appendix A) and all statements are in line with the theoretical framework and were built around the following five variables: the relationships with older people; the thought and beliefs about aging; the feelings about aging; healthy habits followed by Generation Z; and knowledge about aging among Generation Z. The descriptive statistics of the sample are illustrated in Table 5. We notice that most respondents were females (more than 60%), living in urban regions (75%), with a bachelor’s degree (85%), not employed (more than 70%) and still living with their families (65%).

**Table 5:** Descriptive statistics of the sample.

Characteristics		%
Sex	Male	35.88
	Female	64.12
Living environment	Urban	75.95
	Rural	24.05
The last level of studies	High school	6.87
	Bachelor	85.5
	Master	6.11
	PhD	1.52
Employment status	Yes	26.34
	No	73.66
Living alone or with others	Alone	7.25
	With family	65.27
	With other colleagues/friends	14.12
	With partner	13.35

## 4 Results

**Figure 2:**
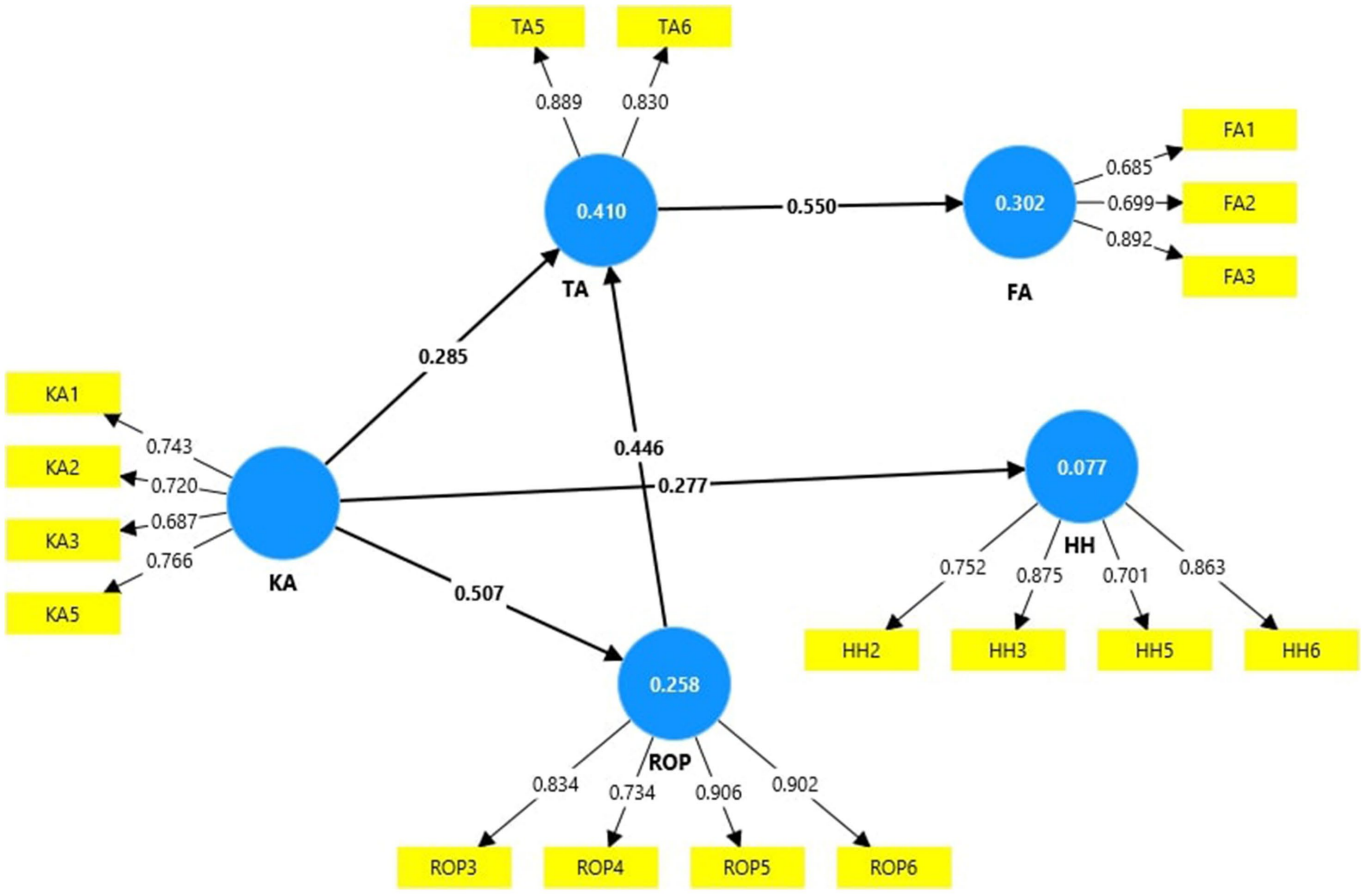
Path coefficients of the model after removing items with lower outer loadings.

Outer loadings and the variance inflation factor (VIF) for the items in the research model are presented in Table 1 as indicators of their reliability and collinearity. All items that do not have outer loadings above 0.6 were removed from the model. Figure 2 presents the research model with the items having good reliability. All VIF values are lower than 5, which shows the model’s collinearity.

The impact of TA on FA (0.550) is the strongest, followed by the impact of KA to ROP (0.507) and from ROP to TA (0.446). A lower impact is registered from KA to TA (0.285) and from KA to HH (0.277). KA and ROP determine 41% of the TA variance; TA generates 30.2% of FA variance; KA generates 25.8% of ROP variance; and KA explains 7.7% of HH variance.

With the PLS-SEM algorithm, the constructs’ reliability and validity have been determined, as shown in Table 6. Cronbach’s alpha for HH, KA and ROP are higher than 0.7, and the values for FA and TA are close to this threshold (higher than 0.65) values considered acceptable (79–83). The composite reliability has high values: most of the constructs register values above 0.7 (84, 85) and TA has a value close to this level (0.669). Thus, the model’s internal consistency is ensured. All values for the average variance extracted (AVE) are higher than 0.5, which shows the model’s validity (73, 86).

**Table 6:** Construct reliability and validity.

Constructs	Cronbach’s alpha	Composite reliability (rho_a)	Composite reliability (rho_c)	Average variance extracted (AVE)
FA	0.698	0.914	0.806	0.585
HH	0.813	0.840	0.877	0.642
KA	0.716	0.729	0.820	0.532
ROP	0.867	0.888	0.910	0.717
TA	0.652	0.669	0.850	0.740

Table 7 presents the use of the Fornell-Larcker criterion for checking the model’s discriminant validity. The main diagonal values are highest in the same column, showing the heterogeneity of the model’s constructs and thus ensuring its discriminant validity.

**Table 7:** Fornell-Larcker criterion.

	FA	HH	KA	ROP	TA
FA	0.765				
HH	0.153	0.801			
KA	0.504	0.277	0.729		
ROP	0.594	0.198	0.507	0.847	
TA	0.550	0.170	0.512	0.591	0.860

The bootstrapping test was applied to check the statistical significance of the model, and the results are presented in Table 8. All values for t statistics are above 1.96, the *p* values are lower than 0.05 and the confidence intervals bias corrected do not include the zero value. Thus, all five hypotheses (H1–H5) are validated.

**Table 8:** The bootstrapping test.

	Path coefficients	T statistics	*p* value	Confidence intervals bias corrected	Hypotheses validation
KA→HH	0.277	5.277	0.000	(0.162, 0.369)	H1 is validated
KA→ROP	0.507	11.057	0.000	(0.407, 0.589)	H2 is validated
KA→TA	0.285	4.458	0.000	(0.156, 0.407)	H3 is validated
ROP→TA	0.446	8.052	0.000	(0.334, 0.552)	H4 is validated
TA→FA	0.550	13.438	0.000	(0.460, 0.621)	H5 is validated

Descriptive statistics for the items kept in the research model are illustrated in Table 2. The lowest means are for FA2 (2.89) regarding aging like their grandparents, HH2 (2.78) related to engagement in sports activities and ROP4 (2.98) is related to the willingness to volunteer for old people. The highest values are registered for KA1 (3.94) regarding the knowledge about the health challenges of old people, TA5 (3.82) about their role in a community due to their experience, FA3 (3.76) about the admiration felt toward old people as role models and KA2 (3.75) regarding knowledge about the challenges in their social lives.

Following the statistical results of our study, we can emphasize that the empirical findings in our study show the positive impact of knowledge about aging on: Generation Z’s habits related to their health; the quality of their relations with their grandparents and other elders; their beliefs on aging and what this process implies. Another finding reflects the impact of Generation Z thoughts and beliefs on aging on their feelings toward aging with the former being influenced by the strong ties they have with old people (grandparents, other family members or elders in the community). The results are presented in more detail in the following Discussion section where we highlight other studies that reached similar conclusions in different cultures.

## 5 Discussion

The present study validated all five hypotheses, showing that education on aging as a process influences the thoughts and beliefs of generation Z regarding old people, but also their habits for a healthy life and the way they interact with elders. Thoughts were also noticed to be influenced by the contacts youngsters have with old people, while at the same time they shaped their feelings toward aging. In this section, we will discuss the following five hypotheses tested in our model.

H1, according to which knowledge about aging has a direct and positive impact on the healthy habits of generation Z was validated, in accordance with other studies in the literature (40, 87). Mirowsky and Ross (87) emphasize that “education develops abilities that help individuals gain control of their own lives, encouraging and enabling a healthy life.” The authors note the significant gaps in elders’ health as a consequence of their education and the fact that they can prevent some diseases if they are healthier and are engaged in physical activities.

H2, according to which knowledge about aging has a direct and positive impact on the relationships with old people, was validated. This is shown by other authors in the literature review (57, 88, 89). Goriup and Lahe (88) conducted a study on how young students are influenced by education on aging and their attitudes in the relationship with older adults change as a consequence. Meshel and McGlynn (89) state that educational programs in which young and older people connect are very efficient in changing their attitudes toward each other. The validation of both H1 and H2 hypotheses points to the need to develop public health and education strategies meant to raise the level of knowledge on aging, which can significantly change the way people interact in communities, at work, in the family, how they age and the control they feel that they have in living their lives.

H3, according to which knowledge about aging has a direct and positive impact on the thoughts and beliefs of generation Z on aging and elders, was validated. This finding is in accordance with other studies in the literature review (40–42, 47). Many authors use terms such as “views,” “perceptions,” “beliefs,” “images,” and “stereotypes” (90) to describe the thoughts and beliefs (which are ingrained thoughts) of younger generations toward old people. Thoughts that are not challenged become beliefs that are more difficult to change, hence the role of education and the need to start it from an early age. Sum et al. (91) note that education reduces the ageism phenomenon among students.

H4, according to which relationships with old people have a direct and positive impact on the thoughts and beliefs about aging held by generation Z, was also validated. Other authors (Gaspar (25, 56–58)) reached similar conclusions. There is a strong link between children and youngsters’ contact with their grandparents or other old adults and their perceptions of aging. Teater (90) also emphasize the role of “intergenerational contact in challenging stereotypes.” Cohn-Schwartz et al. (92) identified two main benefits of contact with older people: more positive views of elders and also more positive perception of respondents’ own aging in the future. Lytle and Levy (93) show that both intergenerational contact and education on aging positively influence youngsters in reducing ageism. Hale (94) notes that relationships between youngsters and elders lead to “lower stereotype scores.”

H5, according to which thoughts about aging have a direct and positive impact on the feelings of generation Z about the aging process and old people, was validated. This hypothesis has its roots in Rational Emotive Behavioral Therapy, which states that dysfunctional or negative views/perceptions/beliefs at a cognitive level influence our emotions and, in the end, our behaviors. Anderson and Gettings (20) use the words “old age scares me,” showing that specific beliefs and views on aging lead to these feelings of fear regarding the aging process. Yao Jr (95) show that expectations (formed in the mind as thoughts and beliefs) lead to a subjective feeling of aging, the authors using the terms “youthful seniors” and “older adult seniors.”

Our research covers the gap in the literature by providing a complex model with 5 variables that are closely connected: thoughts and feelings on aging, healthy habits, knowledge on aging and relations with elders. Other studies consider two or three of these variables but there are no other studies presenting a model that takes into account all of these factors. The firsts three variables are linked to the rational emotive behavior therapy founded by Albert Ellis who highlighted the fact that our behaviors are determined by our thoughts and emotions and if we can change thoughts (including opinions and beliefs), we can change feelings and as a consequence our behaviors.

The findings in our study reveal the importance of knowledge on aging and of developing strong relationships between elders and youngsters. This is useful for shaping public health strategies and making them prioritize both awareness campaigns on aging and programs in which Generation Z has the opportunity to connect with older people on a significant level. Other research papers (96, 97) presented such programs meant to bring benefits for both generations and we appreciate that research on aging, ours included, has the potential to improve results in this field. There are many directions for future research that can be considered: extending the model to Generation Y for which we already started collecting data, but also replicating the model for other countries to compare results and better understand how the similarities or differences are connected to the public health strategies in those countries.

## 6 Conclusion

The present study is focused on generation Z because they represent youngsters who still have the time to change their views and have the maturity to better understand aging as a process. The results show that thoughts and beliefs on aging are shaped by education and intergenerational contact with old people. These findings are useful for developing public health strategies focused on educating people on the changes that occur naturally with age, as well as on the lifestyle choices that can be incorporated into someone’s life in order to age in a healthy way.

Theoretical and practical implications refer to the need for more research on this topic, that can be used by practitioners (public officials, managers in schools and hospitals) to implement the changes required for helping younger generations have a better outlook on aging (in general and their own aging) and old people. This has many implications from a socio-economic and cultural perspective because youngsters can learn from the elders’ experience and thus enrich their working environments and the community’s culture.

Policy and local decision-makers might create opportunities and environments that enable intergenerational interaction. It is within their power to develop national media campaigns presenting old age positively. So far, old age is present in the media through advertisements for medicines, prostheses, or life insurance. Policymakers have the tools to develop media campaigns about the importance of a healthy lifestyle for increasing life expectancy.

Local decision-makers and hospital managers might collaborate to develop public health strategies, including volunteering programs in older adult care centers in villages and towns where young people could help older people, as technology can bridge generations. These volunteering programs might represent a mutual exchange between old and young people. Older people need young people to set up their smartphones, install banking apps, and check their investment accounts. Generation Z might learn confidentiality and reflect on saving money for difficult times. Also, in these volunteering programs, young people could spend a few hours a day with older people, take them for a walk, provide an exercise program, organize their medication or chat with them. Generation Z could thus learn how to communicate at different levels and develop their team spirit, listen and reflect on the stories of older people about their life and professional experiences full of lessons, and the history through the cultural and social transfer from one generation to the next.

The skills obtained from volunteering programs might help generation Z become more attractive to employers. In Hungary, a country close to Romania, volunteering among generation Z subjects, who are significant in the labor market, participation in volunteering activities is related to individual values (personal and skills development). If generation Z’s participation is low in these types of programs, it is due to immaturity or poor information at the individual level or because there are no such opportunities (98).

Romania follows the global urbanization trend, according to Orta Mascaró (99). In Romania, many older adult still live in rural areas, and the proportion of the rural population is still high compared to the European ones (55.6% urban in Romania—44.6% rural). One of Romania’s biggest challenges will be to create and maintain jobs in rural areas (100). Local decision-makers should find solutions to make these jobs attractive for generation Z.

Limitations of the study are related to the geographic limitation, which is focused on generation Z in Romania. Ayalon (67) mentioned the similarities of European countries in the way youngsters perceive elders. Even though this research is limited geographically, the results are still useful considering Romania’s cultural background and post-communist history. As future research directions, we are interested in extending the study to Generation Y and its perceptions on aging, considering that the literature review revealed differences in perceptions between younger and older adults.

## Data Availability

The raw data supporting the conclusions of this article will be made available by the authors, without undue reservation.

## Appendix A

The model’s constructs and items.

**Table:**

Constructs	Items	Codes
Thoughts about aging (TA)	Aging is a process depending on each individual’s choices during his/her life.	TA1
	I think a lot about aging and what this process implies.	TA2
	I have clear opinions about aging and old people (over 65 years old)	TA3
	I think the aging process is naturally associated with disease.	TA4
	Old people are useful for the society because of the experience they gathered during their lives.	TA5
	As an employee or future employee, I think I have many valuable things to learn from old people (over 65 years old)	TA6
Feelings about aging (FA)	I would like to age the same way as my parents did.	FA1
	I would like to age the same way as my grandparents did.	FA2
	I admire many old people who are real role models for me.	FA3
Healthy habits (HH)	I frequently engage in volunteering activities.	HH1
	I regularly engage in sports activities.	HH2
	I pay attention to the way I eat, focusing on healthy choices.	HH3
	I have an active social life.	HH4
	I have hobbies that I practice regularly.	HH5
	I have a healthy lifestyle in general.	HH6
Knowledge about aging (KA)	I am informed about the health challenges of old people.	KA1
	I am informed about the challenges of old people regarding their social life.	KA2
	I am curios and want to know more about the potential problems specific to aging in order to be prepared for this stage of life.	KA3
	I have enough knowledge about aging and the natural changes happening in the body.	KA4
	More knowledge about aging can help improve the quality of my life.	KA5
Relationships with old people (ROP)	The old people I know shared positive experiences with me regarding aging.	ROP1
	I have frequent contact with old people.	ROP2
	Meeting with old people is a pleasurable activity.	ROP3
	I like to volunteer in activities dedicated to old people	ROP4
	I like talking to old people.	ROP5
	When I am around old people, I feel at ease.	ROP6
